# Medical Society of the County of Erie

**Published:** 1896-01

**Authors:** Franklin C. Gram


### Society Proceedings.

### MEDICAL SOCIETY OF THE COUNTY OF ERIE.

Reported by FRANKLIN C. GRAM, M. D., Secretary.

### IN MEMORY OF DR. MAHLON BAINBRIDGE FOLWELL.

A SPECIAL meeting of the Medical Society of the County of Erie was held at the rooms of the Buffalo Academy of Medicine, Wednesday evening, December 11, 1895.

In the absence of the president and vice-president, Dr. John Cronyn was called to the chair.

Dr. Cronyn announced the death of Dr. M. B. Folwell, and stated that this meeting had been called for the purpose of taking appropriate cognisance of the melancholy event, for while it is the duty of the profession to cure others, they must themselves in course of time fall victims to the inevitable.

Dr. J. W. Putnam moved the appointment of a committee to draft a suitable memorial.

The Chair appointed Drs. H. R. Hopkins, D. W. Harrington and F. W. Abbott.

The committee presented the following memorial and moved its adoption :

### MEMORIAL OF DR. MAHLON BAINBRIDGE FOLWELL.

WHEREAS, It has pleased our Heavenly Father to take from us in the prime of life and at the height of a most honorable and useful career, our esteemed colleague, Mahlon Bainbridge Folwell, we, the members of the Medical Society of the County of Erie, recognising that during a period of nearly thirty years Dr. Folwell has been a most valued and efficient member of this society, one who in every act of his professional life was most zealous of its honor and eager for the advancement of the profession which this society represents, wish to bear witness to his character—as a student, most patient and modest ; as a teacher, wise and conservative ; as a practitioner, untiring and skilful.

We tender to his bereaved family our most heartfelt sympathy, and we commend the example of his life as worthy of emulation. While we bow to the decree of an all-wise Providence we mourn his loss as irreparable.

HENRY R. HOPKINS,
DEVILLO W. HARRINGTON,
FRANK W. ABBOTT.

Dr. PUTNAM, in seconding the motion, spoke of the good qualities which the deceased possessed.

Dr. HOPKINS said he had known Dr. Folwell for thirty years, and related the circumstances of their first meeting. When Dr. Hopkins entered the Buffalo Medical College as a student he seated himself on one of the upper seats of the amphitheater. A young man introduced himself as Mr. Folwell and asked permission to occupy the next seat. On that day an acquaintance sprang up which continued in the closest relationship until death separated them. He went to him for advice on all important matters, and he acknowledged that many mistakes would not have been made had he followed his advice. Dr. Folwell was possessed of a soundness and integrity of character which nothing could warp or pervert. If a question was right the cost cut very little figure with him. No man honored his profession more, no man labored harder to keep it bright.

Dr. HARRINGTON spoke of his student days, of his army life and of his graduation in medicine, in 1867, and paid this tribute to him : "A braver soldier, a more conscientious physician and a more loyal friend never lived."

Dr. ABBOTT was too deeply moved to give proper expressions of his feelings. He had known the deceased since his student days and loved him better than anyone except his own family.

Dr. Lucien Howe said that the advisability of holding such meetings as these had been questioned. He thought that the tributes just paid so feelingly by friends answered that argument. We ought to meet to pay proper respect to members who fall in the ranks. Dr. Folwell was a good type of what was one of Nature's gentlemen and a true family physician. He recalled the days when together with the departed and others they sat in the rear rows at the meetings of the profession while the front seats were occupied by men whose names we now revere. Then the middle rows moved forward and the others took second place. In time these dropped out, and now the seats in the front rows are again becoming vacant.

The memorial was adopted and the society adjourned.

---

